# Radiomics for the Prediction of Epilepsy in Patients With Frontal Glioma

**DOI:** 10.3389/fonc.2021.725926

**Published:** 2021-11-22

**Authors:** Ankang Gao, Hongxi Yang, Yida Wang, Guohua Zhao, Chenglong Wang, Haijie Wang, Xiaonan Zhang, Yong Zhang, Jingliang Cheng, Guang Yang, Jie Bai

**Affiliations:** ^1^ Department of MRI, The First Affiliated Hospital of Zhengzhou University, Zhengzhou, China; ^2^ Shanghai Key Laboratory of Magnetic Resonance, East China Normal University, Shanghai, China

**Keywords:** radiomics, glioma, glioma-associated epilepsy, frontal lobe epilepsy, T2 fluid-attenuated inversion recovery

## Abstract

**Objective:**

This study was conducted in order to investigate the association between radiomics features and frontal glioma-associated epilepsy (GAE) and propose a reliable radiomics-based model to predict frontal GAE.

**Methods:**

This retrospective study consecutively enrolled 166 adult patients with frontal glioma (111 in the training cohort and 55 in the testing cohort). A total 1,130 features were extracted from T2 fluid-attenuated inversion recovery images, including first-order statistics, 3D shape, texture, and wavelet features. Regions of interest, including the entire tumor and peritumoral edema, were drawn manually. Pearson correlation coefficient, 10-fold cross-validation, area under curve (AUC) analysis, and support vector machine were adopted to select the most relevant features to build a clinical model, a radiomics model, and a clinical–radiomics model for GAE. The receiver operating characteristic curve (ROC) and AUC were used to evaluate the classification performance of the models in each cohort, and DeLong’s test was used to compare the performance of the models. A two-sided *t*-test and Fisher’s exact test were used to compare the clinical variables. Statistical analysis was performed using SPSS software (version 22.0; IBM, Armonk, New York), and *p <*0.05 was set as the threshold for significance.

**Results:**

The classification accuracy of seven scout models, except the wavelet first-order model (0.793) and the wavelet texture model (0.784), was <0.75 in cross-validation. The clinical–radiomics model, including 17 magnetic resonance imaging-based features selected among the 1,130 radiomics features and two clinical features (patient age and tumor grade), achieved better discriminative performance for GAE prediction in both the training [AUC = 0.886, 95% confidence interval (CI) = 0.819–0.940] and testing cohorts (AUC = 0.836, 95% CI = 0.707–0.937) than the radiomics model (*p* = 0.008) with 82.0% and 78.2% accuracy, respectively.

**Conclusion:**

Radiomics analysis can non-invasively predict GAE, thus allowing adequate treatment of frontal glioma. The clinical–radiomics model may enable a more precise prediction of frontal GAE. Furthermore, age and pathology grade are important risk factors for GAE.

## Introduction

Glioma-associated epilepsy (GAE) is a common diagnosis of glioma patients, which may be attributed to several factors, including tumor location, peritumoral edema, genetic background, and alterations in the microenvironment (1–3). Currently, there is no broadly acknowledged method for GAE interpretation. The frontal lobe is the most common glioma location associated with epilepsy (4), and frontal glioma is the most common cause for frontal lobe epilepsy (FLE) with lesions (5). FLE impairs the recognition and comprehension of patients, and particularly for frontal GAE, if patients do not receive treatment, there is a risk for status epilepticus (5, 6). Early prediction, increased awareness, and proper treatment of GAEs are vital to protect neurocognitive function and improve the quality of life of the patients (7, 8). With respect to the location of epileptic discharges, the propagation mode, and the experience of the patients, FLE differs from epileptic discharges in extra frontal regions (5); therefore, we independently analyzed frontal GAE in this study.

Radiomics is an emerging method to obtain predictive or prognostic information from medical images by several quantitative image features (9, 10). Although such features are not directly apparent to clinical practitioners, these can potentially produce reliable diagnostic and prognostic models in conjunction with other information sources. Radiomics has been proven helpful for clinical diagnosis, treatment choice, and prognosis assessment based on tomographic imaging (computed tomography, CT; magnetic resonance imaging, MRI; and positron emission tomography, PET) (11–14).

MRI is an essential preoperative examination for patients with glioma, and radiomics based on MRI is one of the focus areas for glioma research. The MRI sequences selected for glioma in previous radiomics research were mostly based on conventional MRI sequencing, such as T2-weighted imaging (T2WI), T2 fluid-attenuated inversion recovery (T2 FLAIR) imaging, apparent diffusion coefficient maps, and contrast-enhanced T1-weighted sequences (15–20). Given its high spatial resolution and the fact that it does not require materials, T2 FLAIR MRI sequences are considered to provide more tumor information than T2WI in glioma research (21). Radiomics analysis in T2WI has been previously applied to GAE (22–24) and has proven effective to predict the occurrence and type of GAE. However, these studies focused on low-grade glioma (LGG) and neglected the influence of brain tumor location on the seizure propagation mode. Therefore, we used the radiomics features extracted from T2 FLAIR imaging to investigate the association between radiomics features and WHO II–IV grade frontal lobe gliomas concurrent with epilepsy.

## Material and Methods

### Patients and Magnetic Resonance Imaging

**Figure 1:**
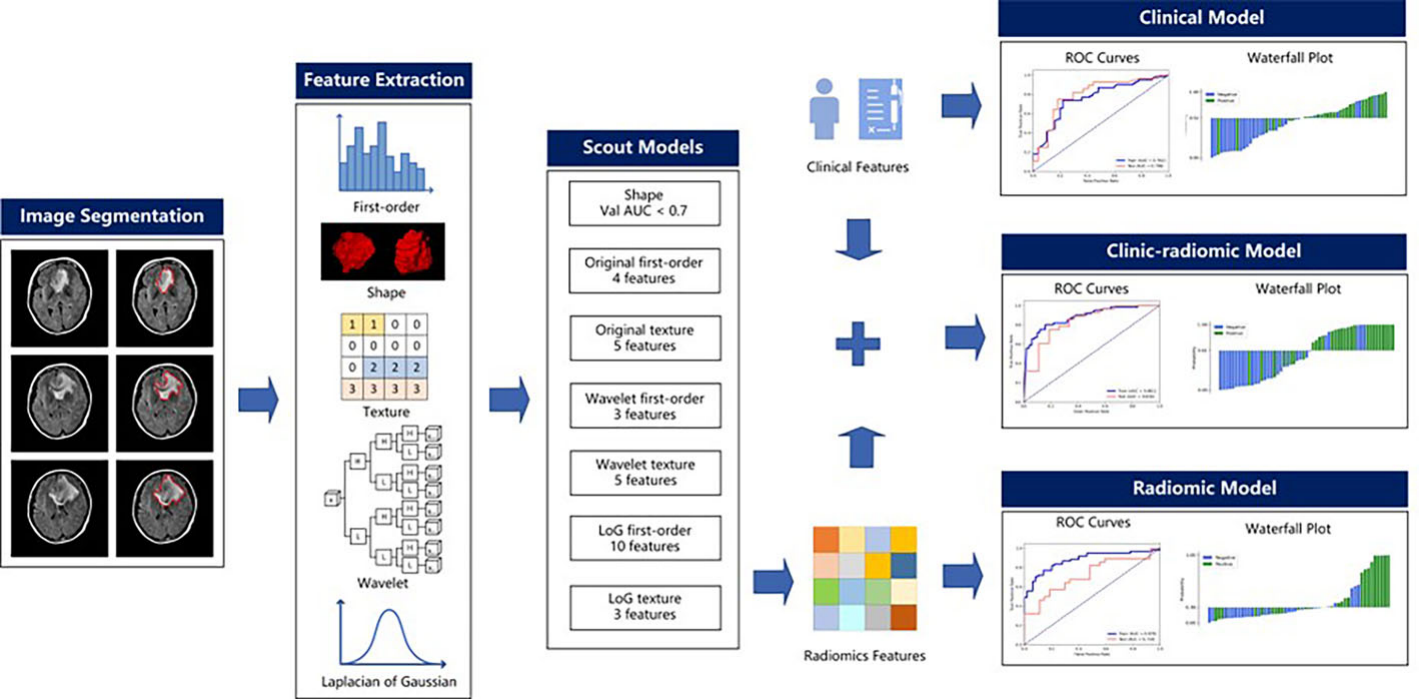
Radiomics workflow.

This retrospective study was approved by the Ethics Committee of Scientific Research and Clinical Experiments of the First Affiliated Hospital of Zhengzhou University, which waived the requirement for written consent. We enrolled 166 consecutive patients with frontal glioma who underwent MRI scanning before surgery at our hospital from August 2016 through August 2019. Inclusion criteria were a) a single tumor and peritumoral edema defined in the frontal lobes by neurosurgery and imaging findings, b) pathologically confirmed single frontal gliomas (WHO II–IV) according to the 2016 WHO criteria, and c) available presurgical T2 FLAIR imaging data. Exclusion criteria were a) satellite tumors of the frontal glioma located beyond the frontal lobe and WHO grade I glioma, b) patients who underwent puncture biopsy and started antitumoral therapy before the MRI scan, and c) frontal glioma patients with other recent lesions that could cause epilepsy, such as cerebral hemorrhage, stroke, and other brain tumors. The preoperative diagnosis of GAE was based on clinical signs, electroencephalography (EEG), and imaging findings (25). According to clinical preoperative diagnosis, the enrolled cases included 89 patients with epilepsy and 77 patients without. The dataset was randomly split into a training cohort (*n* = 111, epilepsy/no epilepsy = 61/50) and a test cohort (*n* = 55, epilepsy/no epilepsy = 28/27). The sex and age of the patients, the tumor grade, and tumor location (left/right/both) were recorded. The whole workflow is illustrated in **Figure 1**.

MR images scanned with 3.0 T MRI scanners (Magnetom Trio TIM/Prisma, Verio or Skyra, Siemens Healthcare; Discovery 750, GE Medical Systems) were retrieved from the Picture Archive and Communication System of the hospital. MRI T2 FLAIR images had a 256 × 256-pixel matrix and a 240 × 240-mm field of view, inversion time = 2,400–2,500 ms, echo time = 81–135 ms, and repetition time = 8,000–8,500 ms, with section thickness = 5 mm and intersection gap = 1 mm.

### Tumor Masking and Image Preprocessing

One volume of interest (VOI) including the entire tumor and peritumoral edema was manually drawn slice-by-slice on T2 FLAIR images using the ITK-SNAP (version 3.6.0; www.itksnap.org) software by a Ph.D. candidate in imaging for medicine (AG, 5 years of experience in neuro-oncology). Next, the segmentation results were reviewed and modified if necessary by a neuroradiologist with 20 years of experience in neuroradiology (JB) using the same software. All images were normalized to a [0, 1] range before feature extraction.

### Radiomics Feature Extraction

Feature extraction was performed with the PyRadiomics (version 3.0) (26) package in Python (3.7.6). For each case, 3D shape features (*n* = 14) were extracted from the VOI before first-order statistics features (*n* = 18); texture features (*n* = 75) were extracted from each of the following image types: 1) original T2 FLAIR images, 2) each of the three Laplacian-of-Gaussian (LoG) filtered images (sigma = 1.0, 3.0, 5.0), and 3) each of the eight sub-bands of 3D wavelet-transformed image sub-bands using the Haar wavelet. The texture features extracted in this study included features based on a gray-level co-occurrence matrix (GLCM), gray-level run length matrix (GLRLM), gray-level size zone matrix (GLSZM), neighboring gray tone difference matrix (NGTDM), and gray-level dependence matrix (GLDM). A total of 1,130 features were extracted for each case.

### Feature Selection and Model Building

To remove the imbalance from the training dataset, upsampling by repeating random cases was applied to balance the GAE and non-GAE group samples.

A total of 1,130 radiomics features were extracted for each case; however, dealing directly with such an extensive feature number limits the robustness and effectiveness of the model. Thus, to reduce the dimensions of the features and select the appropriate ones for radiomics model building, we used a heuristic approach. First, all features were divided into subgroups according to their category, such as first-order, shape, and texture, and a scout model with those features was built for each subgroup using the training dataset. The scout models were evaluated with a 10-fold cross-validation; the optimal model in each subgroup was selected by its performance on cross-validation with the training cohort. When the area under the receiver operating characteristic curve (AUC) of the optimal scout model on cross-validation was >0.7, all features in the model were used for the final model building. Otherwise, no subgroup features were further used.

To build the scout model, all features were normalized to the [0, 1] range. Thereafter, Pearson correlation coefficient (PCC) values between all feature pairs were calculated, and if the PCC value between two features was >0.99, one of them was removed. To determine the best number of features to be retained in the model, three feature selectors were compared: recursive feature elimination (RFE), which repeatedly builds the model and eliminates the least important feature; relief, which calculates a feature score for every feature and ranks them accordingly; and the Kruskal–Wallis test (KW), which eliminates the most likely feature from the same distribution in both GAE and non-GAE samples. For the classifier, we compared the performance of linear support vector machine (SVM), logistic regression (LR), and random forest (RF) for their good interpretability and demonstrated good performance in diagnosis based on medical imaging.

To find the best model, we tested different combinations of feature selectors and classifiers. Therefore, nine models (three features selectors and three classifiers) were built for a scout model, and the one with the best cross-validation AUC was used. Multimodel building and comparison were implemented semiautomatically with an open-source software, FeAtureExplorer (FAE, version 0.3.3) (27), which uses scikit-learn (version 0.23.2) as backend for machine learning.

As mentioned above, the features retained in qualified scout models were used to build a radiomics model. We also established a clinical model using only clinical variables (sex, age, tumor grade) and a clinical–radiomics model using both selected radiomics features and clinical variables to distinguish GAE from non-GAE. The process for building the above models was similar to those used in scout model building, but without PCC dimension reduction, due to the relatively small number of input features.

### Performance Evaluation of the Models

The receiver operating characteristic curve (ROC) and AUC were used to evaluate the classification performance of the models in each cohort. DeLong’s test was used to observe AUC differences in the models. The accuracy, sensitivity, specificity, positive predictive value (PPV), and negative predictive value (NPV) were also calculated at the cutoff value that maximizes the Youden index value in the training cohort. Calibration curves and decision curve analysis (DCA) (28) served to assess the clinical usefulness of radiomics signatures.

### Statistical Analysis

Age is reported as mean and range, and their difference between the GAE and non-GAE groups was assessed by a two-sided *t*-test. Sex and glioma position and grade were reported as frequency and proportions, and differences between the GAE and non-GAE groups were assessed by Fisher’s exact test. Statistical analysis was performed using SPSS software (version 22.0; IBM, Armonk, New York), and *p <*0.05 was used as the threshold for significance.

## Results

### Demographic and Clinical Data

The main clinical and pathological characteristics of all 166 patients are listed in **Table 1**. There were significant differences between the GAE and non-GAE groups with respect to age, sex, and glioma grade (*p* < 0.05). Thus, younger, male, and LGG patients had a higher risk of GAE. Age distribution and glioma subtypes in the full cohort of patients are listed in **Appendix 1**.

**Table 1:** Clinical characteristic of patients in the training and testing cohorts.

Characteristics	All cohort (*n* = 166)		*p*-value	Training cohort (*n* = 111)		*p*-value	Testing cohort (*n* = 55)		*p*-value
	Non-GAE group	GAE group		Non-GAE group	GAE group		Non-GAE group	GAE group	
Sample size	77	89	–	50	61	–	27	28	–
Male/female	32/45	60/29	0.001	24/26	43/18	0.020	9/19	17/10	0.031
Age mean ± SD (range)	49 ± 12 (15–74)	41 ± 12 (12–66)	<0.001	48 ± 11 (15–73)	41 ± 12 (12–65)	0.005	50 ± 12 (25–74)	41 ± 12 (17–66)	0.014
Glioma position (left/right/both)	30/26/21	35/45/9	0.009	21/16/12	25/30/7	0.107	8/10/9	10/16/2	0.051
Glioma grade (WHO II/III/IV)	22/16/39	56/19/14	<0.001	16/10/24	38/13/10	0.001	7/6/14	18/6/4	0.006

p-values of age are the results of independent-samples t-tests; p-values of gender and tumor grade are the results of Fisher’s exact tests.

In the GAE group (89/166), 18 patients (18/89) had preoperative EEG [ambulatory EEG (*n* = 10), video EEG (*n* = 6), conventional EEG (*n* = 1), and intracranial EEG (*n* = 1)], and two of them (2/18) showed interictal epileptiform abnormalities (IEAs) in the EEG recording; the others (16/18) had a normal EEG recording. All patients in the non-GAE group had no EEG recordings.

### Performance of the Models

To find valuable features to build the radiomics model, we divided all radiomics features into seven categories and built a scout model for each. Using the features retained in scout models, we built a final radiomics model. Additionally, a clinical model using clinical variables and a clinical–radiomics model using both radiomics features and clinical variables were built. All models and features are listed in **Appendix 2**.

**Figure 2:**
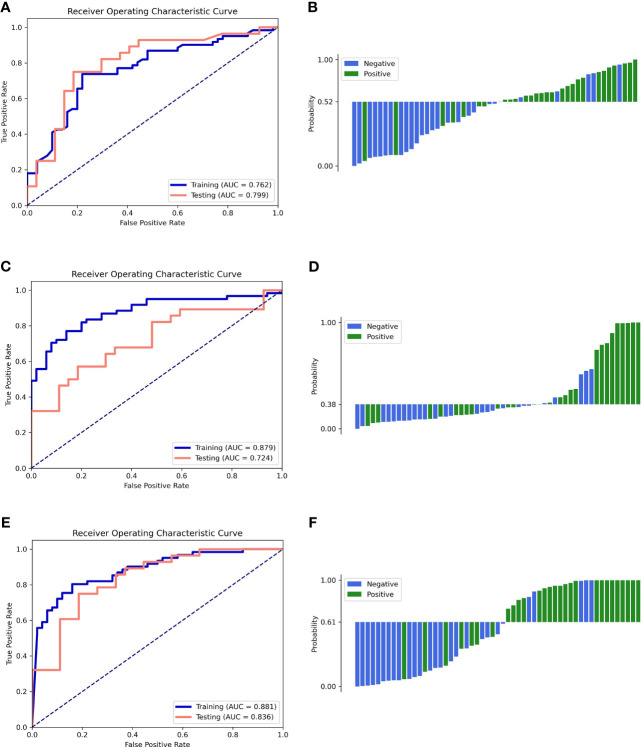
ROC curves of the training and testing cohorts (left column) and the waterfall plot of the distribution of prediction probability on the testing cohort (right column). **(A, B)** Clinical model. **(C, D)** Radiomics model. **(E, F)** Clinical–radiomics model.

**Figure 3:**
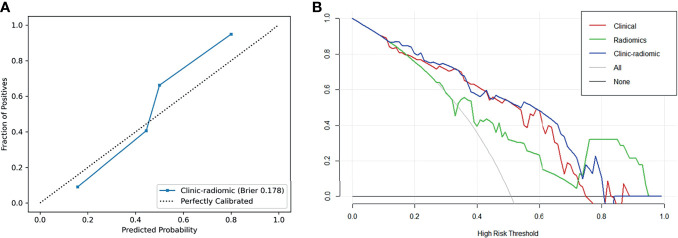
**(A)** Calibration curve of the clinical–radiomics model. **(B)** DCA curves of the clinical, radiomics, and clinical–radiomics model.

**Figure 4:**
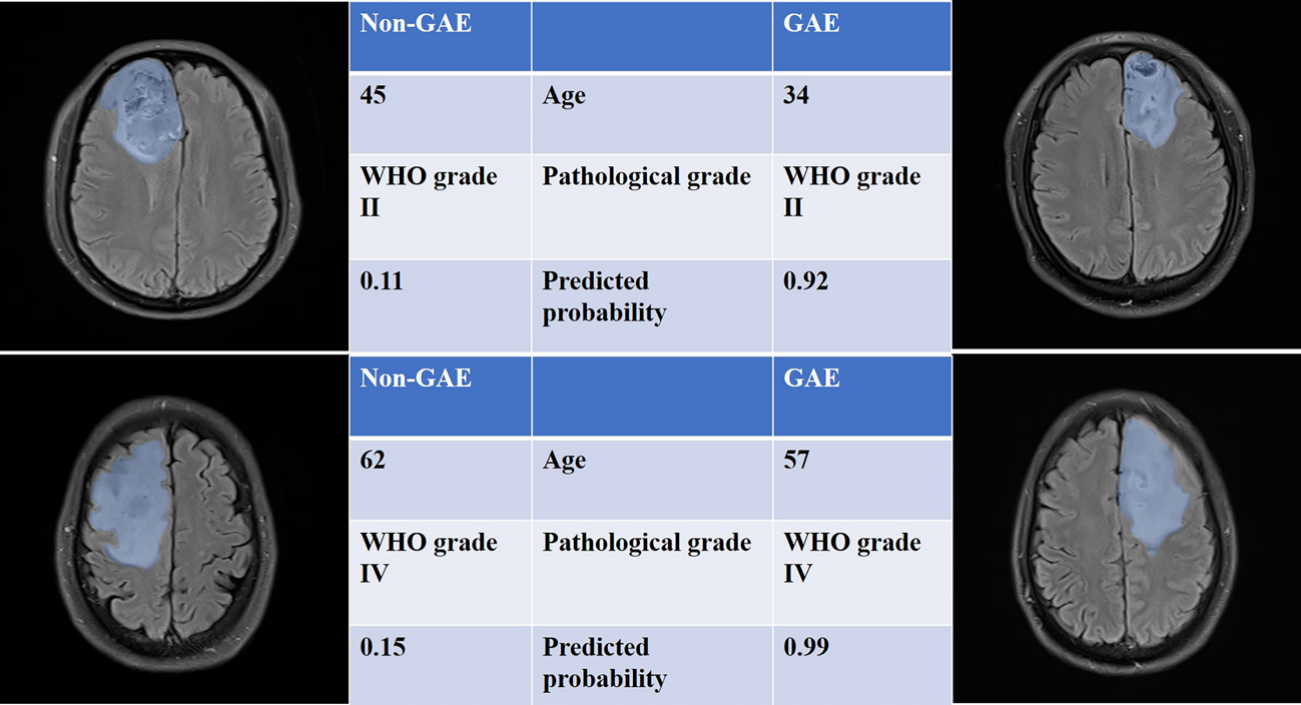
T2 FLAIR images of four patients with or without GAE experience. The blue shadow in the images was manually delineated as the region of VOI. All the clinical characteristics and predicted probabilities of the combined model are presented at the center of the table.

The performance of all models is listed in **Table 2**. The ROC curves and the predicted probability of clinical, radiomics, and clinical–radiomics models are shown in **Figure 2**. The classification accuracy of the scout models was <0.75 in cross-validation, except the wavelet first-order and wavelet texture models. Among all models, the clinical–radiomics model including 17 radiomics features and two clinical features achieved a performance with a classification accuracy = 0.82 and AUC = 0.886 [95% confidence interval (CI), 0.819–0.940] in the training cohort and a classification accuracy = 0.782 and AUC = 0.836 (95% CI, 0.707–0.937) in the testing cohort. The clinical–radiomics model achieved the best performance on the testing cohort (**Table 3**). DeLong’s test showed a *p*-value <0.05 (*p* = 0.008) between the radiomics and clinical–radiomics models (**Table 4**). The calibration curve and DCA for the clinical–radiomics model are shown in **Figures 3A, B**, and its calibration performance, as evaluated with the Brier score, is reported in the legend. **Figure 4** shows a comparison between two representative patient cases with similar image and clinical representation; the clinical–radiomics model effectively distinguished between individuals with GAE and without GAE among glioma patients; the DCA curve revealed that for a high-risk threshold between 0.1 and 0.7, the clinical–radiomics model can be more beneficial than the clinical and radiomics models.

**Table 2:** The performance of all models in predicting GAE in the training and testing cohorts.

Model	Cohort	AUC (95% CI)	Accuracy	Sensitivity	Specificity	PPV	NPV
Clinical model	Training	0.762 (0.667–0.846)	0.748	0.721	0.780	0.800	0.696
	Testing	0.799 (0.672–0.917)	0.782	0.750	0.815	0.808	0.759
Radiomics features-combined model	Training	0.879 (0.805–0.939)	0.811	0.770	0.86	0.870	0.754
	Testing	0.724 (0.575–0.855)	0.673	0.536	0.815	0.750	0.629
Clinical–radiomics model	Training	0.886 (0.819–0.940)	0.820	0.803	0.840	0.860	0.778
	Testing	**0.836 (0.707**–**0.937)**	0.782	0.750	0.815	0.808	0.759

In the process of establishing scout models to select features, only the cross-validation performance was assessed to avoid information leakage. The bold values is optimal value.

AUC, area under the curve; PPV, positive predictive value; NPV, negative predictive value.

**Table 3:** Selected features and the coefficients of features in the clinical–radiomics model.

Features	Coefficients of SVM
Wavelet HHL GLCM correlation	2.109668663
Wavelet LHL GLCM correlation	1.729482221
Wavelet LHL GLRLM run variance	1.691610793
Wavelet HHL GLDM large dependence low gray-level emphasis	1.618789140
LoG sigma 3.0 mm 3D GLDM dependence non-uniformity normalized	1.536185789
Wavelet HHL GLDM low gray-level emphasis	1.513109907
Wavelet HHL first-order kurtosis	1.398738067
LoG sigma 5.0 mm 3D GLDM dependence non-uniformity normalized	1.375653827
Wavelet HHL first-order 10 percentile	1.356330395
Wavelet HHL first-order root mean squared	−1.347183074
Pathological grade	−1.082656461
Original GLDM high gray-level emphasis	1.032809669
Original GLSZM size zone non-uniformity normalized	−0.836530847
Age	−0.580041869
LoG sigma 1.0 mm 3D GLSZM small area emphasis	−0.444386255
Original first-order mean	0.363086015
Original first-order 90 percentile	0.262011650
Original first-order total energy	−0.189844398
LoG sigma 5.0 mm 3D first-order total energy	−0.089783744

GLCM, gray-level co-occurrence matrix; GLDM, gray-level dependence matrix; GLRLM, gray-level run length matrix; GLSZM, gray-level size zone matrix; HLL, HHL, LHL, HLH, considering L and H to be a low-pass (i.e. a scaling) and a high-pass (i.e., a wavelet) function; LoG, Laplacian-of-Gaussian.

**Table 4:** Comparison of the performance of the models.

Comparison	DeLong’s test* (*p*-value) in the testing cohort	DeLong’s test* (*p*-value) in all cohorts
Clinical model vs. radiomics model	0.456	0.266
Clinical model vs. clinical–radiomics model	0.648	0.014
Radiomics model vs. clinical–radiomics model	0.008	0.047

p-value <0.05 indicated a statistically significant difference. *Test for the comparison of the difference of AUC.

## Discussion

Despite the higher incidence of epilepsy in LGG than in high-grade glioma (HGG) (29, 30), HGG is more common, and epilepsy in HGG patients always suggests tumor progression (31). Therefore, a non-invasive way to predict the occurrence of epilepsy in both LGG and HGG patients is needed to improve care, treatment, and timely surgery. Radiomics, which can transform medical images into useful data, has been widely used to classify glioma grade- or subtype-associated mutations and predict tumor proliferation, patient prognosis, etc. (15–20, 22).

In this study, T2 FLAIR-based radiomics was used to automatically extract 1,130 quantitative features, and the optimal model was selected from 10 models for predicting the occurrence of GAE in glioma patients. The best model, which combined clinical and radiomics features, could distinguish GAE patients from glioma patients with satisfying accuracy. Among all scout models, those based on wavelet transform features exhibited better performance than those based on other features, consistent with the higher weight of wavelet features in the combined models. This suggests the effectiveness of scout models to find useful features. The importance of wavelet features in our models was also consistent with previous research on epilepsy and epilepsy-type prediction in LGG (22, 24). Furthermore, in our study, the features from LoG filtered images were also included and were contributing features in the final models. LoG features are closely related to glioma heterogeneity, tumor microenvironment (10), and personalized tumor information (32) and are widely used in cancer radiomics (11–13).

In the best model, the age of the patients and tumor grade negatively correlated with GAE, which is also consistent with former reports (22, 24). Age is known to be related to a decrease in gamma-aminobutyric acid levels (33), and the metabolism of glioma may increase neuronal activity (7, 34), which implies that older glioma patients are more likely to experience GAE. However, although older patients are more likely to have HGG, LGG is more frequent in younger patients (35, 36). As reported by Englot et al. (1), HGGs have a predilection for white matter, which may preclude epileptogenic development. Furthermore, some old patients with malignant tumors may not survive long enough to develop epilepsy or may have other severe symptoms requiring a visit to a doctor before epilepsy develops. Therefore, younger patients have a higher risk of GAE than older patients. Additionally, LGG has a higher IDH mutation rate than HGG (37). Not only is IDH a vital gene for glioma genotyping (37), but IDH mutation type is also associated with GAE (7). Thus, the influence of the age of the patients and tumor grade in GAE is very complex. SVM coefficients of age and tumor grade were not very large; however, the clinical–radiomics model, which used these two variables, still achieved a higher AUC in the test cohort than the radiomics model. Thus, age and tumor grade are necessary features to improve the predictive efficiency of the model.

Tumor location and tumor cell impact on the peritumoral cortex are important factors for epilepsy occurrence, propagation mode, and subtype (1–4, 38), which are used in the research of epilepsy with lesion as critical categorized data (39). It is hard to accurately describe the glioma location in subregions, as there are many FLE-originating subregions, including motor areas and the cingulate gyrus, frontopolar, orbitofrontal, and dorsolateral cortex (40). Furthermore, most gliomas are ill-defined and irregular in shape and involve >1 brain subregion. Liu et al. (22) used the distance from the anterior commissure to the tumor centroid for a quantitative description of the tumor subregional location (22) and demonstrated that the Chebyshev distance significantly contributed to epilepsy prediction. Although no information on subregional tumor location was used in our research, all tumors were located in the frontal lobe and the VOIs included the peritumoral cortex, and our clinical–radiomics model for frontal GAE prediction achieved a slightly better result than that of Liu et al. (22).

The present study has some limitations. First, the diagnosis of GAE was based on clinical presentation rather than EEG analysis. Most GAE patients have an average EEG performance without seizures, and a short preoperative waiting period increases the difficulty of capturing an effective EEG. Second, IDH genotype was not included among the clinical features. In the future, machine learning methods for preoperative glioma IDH genotype prediction may improve the precision of GAE prediction. Third, a multicenter, large-scale prospective clinical trial is required to address the limitation of the small samples originating from a single institution in the present study.

In conclusion, radiomics analysis can non-invasively predict epilepsy to ensure proper treatment of frontal glioma patients. Our results suggest that the clinical–radiomics model may allow for a more precise GAE prediction in frontal glioma. Furthermore, age and pathology grade are important risk factors for GAE.

## Data Availability Statement

The original contributions presented in the study are included in the article/**Supplementary Material**. Further inquiries can be directed to the corresponding authors.

## Ethics Statement

The studies involving human participants were reviewed and approved by the Ethics Committee of Scientific Research and Clinical Experiments of the First Affiliated Hospital of Zhengzhou University. Written informed consent for participation was not required for this study in accordance with the national legislation and the institutional requirements.

## Author Contributions

AG: manuscript preparation, literature research, data acquisition, statistical analysis, and manuscript editing. HY: manuscript preparation, literature research, data analysis, and statistical analysis. YW: literature research, data analysis, and statistical analysis. GZ, CW, and HW: literature research and data acquisition. XZ and YZ: data analysis and study design. JC, GY, and JB: study concept and design, manuscript review, and guarantor of the integrity of the entire study. All authors contributed to the article and approved the submitted version.

## Funding

This study was supported by a public service platform for artificial intelligence screening and auxiliary diagnosis for the medical and health industry, Ministry of Industry and Information Technology of the People’s Republic of China (No. CEIEC-2020-ZM02-0103/03); National Natural Science Foundation of China (61731009); National Key R&D Program of China (2016YFC0106900); and Medical Tackling Problems in Science and Technology Plain Program of Henan Province, China (201702070).

## Conflict of Interest

The authors declare that the research was conducted in the absence of any commercial or financial relationships that could be construed as a potential conflict of interest.

## Publisher’s Note

All claims expressed in this article are solely those of the authors and do not necessarily represent those of their affiliated organizations, or those of the publisher, the editors and the reviewers. Any product that may be evaluated in this article, or claim that may be made by its manufacturer, is not guaranteed or endorsed by the publisher.
