# Supplementary material for: Radiomics for the Prediction of Epilepsy in Patients With Frontal Glioma
Source: Front Oncol. 2021 Nov 22;11:725926. doi: 10.3389/fonc.2021.725926 (PMC8645689; doi:10.3389/fonc.2021.725926)
Supplement: Supplementary file 1 [file DataSheet_1.pdf]

Appendix 1a. The glioma sub-types in cohort of all patients.

	**WHO grade II (n=78)**		**WHO grade III (n=35)**		**WHO grade IV (n=53)**		**All cohort**	
Group	IDH_W	IDH_M ($1p19q_{non\text{-}codel}$/$1p19q_{codel}$/$1p19q_{unknown}$)	IDH_W	IDH_M ($1p19q_{non\text{-}codel}$/$1p19q_{codel}$/$1p19q_{unknown}$)	IDH_w	IDH_M	IDH_w	IDH_M
GAE group	3	53 (7/28/18)	3	16 (1/10/5)	11	3	17	72
Non-GAE group	3	19 (11/5/3)	1	15 (0/13/2)	35	4	39	38
Age (mean ± SD)	-	-	-	-	-	-	50.5±14.1	42.3±10.9

Note. IDH_W, isocitrate dehydrogenase wild type. IDH_M, isocitrate dehydrogenase mutation type. $1p19q_{non\text{-}codel}$/$1p19q_{codel}$/$1p19q_{unknown}$, 1p19q non-codeletion/1p19q codeletions/1p19q has no pathology result.

Appendix 1b. The age distribution of all patients.

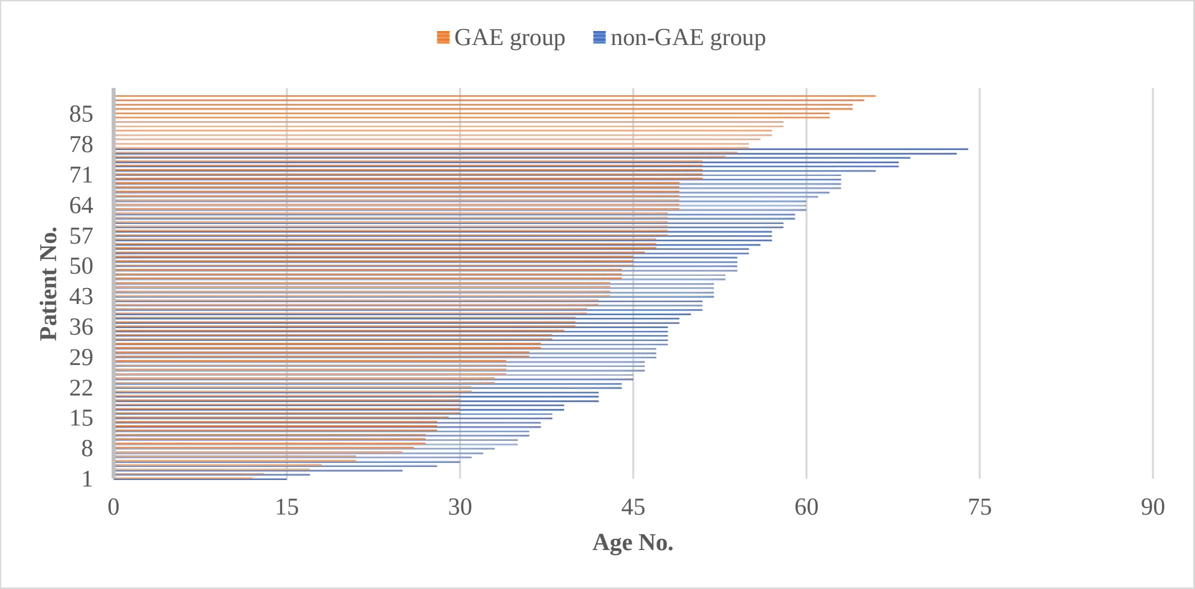

Appendix 2. Features selected in models.

Model	Feature number	Individual features	Feature contribution (P or N)
Shape model	5	Maximum 3D diameter	4.043638915 (P)
		Flatness	-4.771486845 (N)
		Maximum 2D diameter column	5.128985868 (P)
		Maximum 2D diameter slice	-6.839350673 (N)
		Major axis length	-7.554585705 (N)
Original first order model	4	Mean	-3.453293469 (N)
		90 Percentile	-4.983274637 (N)
		Total energy	-23.529752261 (N)
		Energy	23.888512103 (P)
Original texture model	5	GLDM large dependence low grade gray level emphasis	/
		GLCM cluster shade	/
		GLDM high gray level emphasis	/
		GLRLM long run high gray level emphasis	/
		GLSZM size zone nonuniformity normalized	/
LoG first order model	10	5.0 mm variance	1.711433659 (P)
		1.0 mm variance	-2.565389036 (N)
		3.0 mm variance	3.480931693 (P)
		5.0 kurtosis	5.131740485 (P)
		1.0 mm Interquartile range	-5.672505266 (N)
		3.0 mm total energy	-7.025115512 (N)
		1.0 mm total energy	-7.673385179 (N)
		1.0 mm energy	9.899469043 (P)
		5.0 mm total energy	-10.310174754 (N)
		3.0 mm energy	11.859891052 (P)
LoG texture model	3	1.0mm 3D GLSZM small area emphasis	/
		5.0mm 3D GLDM dependence nonuniformity normalized	/

		3.0mm 3D GLDM dependence nonuniformity normalized	/
Wavelet first order model	3	HHL kurtosis	/
		HHL root mean squared	/
		HHL 10 percentile	/
Wavelet texture model	5	HHL GLDM low gray level emphasis	4.606567694 (P)
		HHL GLDM large dependence low gray level emphasis	4.648666650 (P)
		LHL GLCM correlation	9.817323275 (P)
		HHL GLCM correlation	11.179233591 (P)
		LHL GLRLM run variance	16.401634450 (P)
Clinical model	3	Gender	-2.216162957 (N)
		Age	-2.576434188 (N)
		Pathological grade	-3.726135869 (N)
Radiomic features combined model	14	Original first order 90 percentile	-0.098420960 (N)
		Original first order mean	0.157985654 (P)
		Wavelet HHL first order root mean squared	-0.237031026 (N)
		LoG 1.0mm 3D GLSZM small area emphasis	-0.350729256 (N)
		Wavelet HHL GLDM low gray level emphasis	0.355376817 (P)
		LoG 3.0mm 3D GLDM dependence non-uniformity normalized	0.401618639 (P)
		Wavelet HHL GLDM large dependence low gray level emphasis	0.444118604 (P)
		Wavelet HHL first order 10 percentile	0.554403570 (P)
		Wavelet LHL GLRLM run variance	0.763101889 (P)
		Original GLSZM size zone non-uniformity normalized	-0.764766468 (N)
		LoG 5.0mm 3D GLDM dependence non-uniformity normalized	0.876333725 (P)
		Wavelet HHL GLCM correlation	0.885652900 (P)
		Wavelet LHL GLCM correlation	0.968529651 (P)
		Wavelet HHL first order kurtosis	1.084351551 (P)
Clinic-radiomic model	19	The same with Table 3.	

Note. P or N, shortly for positive and negative correlation in model. GLCM, Gray-Level Co-occurrence Matrix; GLDM, Gray Level Dependence Matrix; GLRLM, Gray Level Run Length Matrix; GLSZM, Gray-Level Size Zone Matrix. "/"indicates that the model used random forest as a classifier, so there was no definite contribution of features.

Appendix 3.

All features were normalized before feature selection. To reduce their dimension and select the appropriate ones for radiomics model building, we used a heuristic approach. First, features were divided into sub-groups according to their category, such as first-order, shape and texture, and a scout model was built with those features for each subgroup, using the training dataset. The scout models were evaluated with a 10-fold cross-validation, and when the average cross-validation area under the receiver operating characteristic curve (AUC) was > 0.6, all features in the model were retained for final model building. Otherwise, no subgroup features were further used."

For example, 14 shape features formed a subgroup. In this group, 9 different models pipelines (3x3) were built and comparedperformed as we described before.

Appendix 4 and 5 list the first five models sorted by their performance on cross-validation cohort. Since the optimalbest scout model obtained an AUC of 0.647 which was smaller than 0.7, shape features were not used for establishing radiomics signature and clinic-radiomic model. Conversely, for the subgroup of first-order features in original images, the optimalbest scout model achieved an AUC of 0.758 on cross-validation set, so 4 features retained in this model were selected for the building of radiomics modelsfurther trail. In this way, redundant features were reduced, which was helpful to find candidate model with all categories of features. It is worth noof noteing that the process of feature selection was only performedonly carried on training cohort via cross-validation, so without leaking information leaking of testing cohort was avoided.

Appendix 4. The first five models in each subgroup ordered by their AUC on cross-validation cohort. Model named by its feature selector method, classifier and the feature number it used.

Scout model	Model name	AUC on cross-validation cohort
Shape	Relief-LR-5	0.647
	RFE-LR-2	0.646
	RFE-LR-3	0.642
	RFE-LR-5	0.641
	RFE-LR-4	0.641
Original first-order	Relief-LR-4	0.738
	Relief-LR-10	0.737
	Relief-LR-5	0.736
	Relief-LR-6	0.734
	Relief-LR-11	0.723
Original texture	Relief-RF-5	0.784
	KW-LR-19	0.783
	KW-LR-20	0.782
	KW-LR-18	0.781
	Relief-RF-22	0.780
Wavelet first-order	RFE-RF-3	0.831
	RFE-LR-15	0.826
	RFE-LR-9	0.823
	RFE-LR-12	0.822
	RFE-LR-14	0.821
Wavelet texture	RFE-LR-5	0.828
	RFE-SVM-4	0.824
	RFE-LR-4	0.822
	RFE-SVM-5	0.813
	Relief-SVM-26	0.811
LoG first-order	Relief-LR-10	0.786
	Relief-LR-7	0.785
	Relief-LR-8	0.764
	RFE-LR-16	0.761
	Relief-LR-9	0.759
LoG texture	KW-RF-3	0.817
	KW-RF-2	0.805
	RFE-SVM-13	0.787
	KW-RF-13	0.783
	RFE-SVM-14	0.782

Appendix 5. The performance of scout models in predict GAE in training and testing cohort.

Model	Cohort	AUC (95% CI)	Accuracy	Sensitivity	Specificity	PPV	NPV
Shape model	Training	0.684 (0.577-0.785)	0.694	0.738	0.640	0.714	0.667
	Cross-validation	0.647 (0.538-0.752)	0.658	0.607	0.720	0.736	0.600
Original first order model	Training	0.758 (0.664-0.840)	0.730	0.754	0.700	0.754	0.700
	Cross-validation	0.738 (0.639-0.831)	0.703	0.623	0.800	0.792	0.635
Original texture model	Training	0.923(0.868-0.964)	0.847	0.754	0.960	0.958	0.762
	Cross-validation	0.784 (0.698-0.868)	0.730	0.541	0.960	0.943	0.632
LoG first order model	Training	0.786 (0.698-0.867)	0.748	0.853	0.620	0.732	0.775
	Cross-validation	0.786 (0.698-0.864)	0.730	0.639	0.840	0.830	0.656
LoG texture model	Training	0.733 (0.634-0.828)	0.757	0.574	0.980	0.972	0.653
	Cross-validation	0.817 (0.736-0.891)	0.748	0.623	0.900	0.884	0.662
Wavelet first order model	Training	0.781 (0.692-0.865)	0.730	0.525	0.980	0.970	0.628
	Cross-validation	0.831 (0.747-0.903)	0.793	0.754	0.840	0.852	0.737
Wavelet texture model	Training	0.815 (0.729-0.889)	0.784	0.689	0.90	0.894	0.703
	Cross-validation	0.828 (0.739-0.897)	0.784	0.738	0.840	0.849	0.724
Clinical model	Training	0.762 (0.667-0.846)	0.748	0.721	0.780	0.800	0.696
	Testing	0.799 (0.672-0.917)	0.782	0.750	0.815	0.808	0.759
Radiomic features combined model	Training	0.879 (0.805-0.939)	0.811	0.770	0.86	0.870	0.754
	Testing	0.724 (0.575-0.855)	0.673	0.536	0.815	0.750	0.629
Clinic-radiomic model	Training cohort	0.886 (0.819-0.940)	0.820	0.803	0.840	0.860	0.778
	Testing	**0.836 (0.707-0.937)**	0.782	0.750	0.815	0.808	0.759
Model	Cohort	AUC (95% CI)	Accuracy	Sensitivity	Specificity	PPV	NPV
	Cross-validation	0.647 (0.538-0.752)	0.658	0.607	0.720	0.736	0.600
Original first order model	Training	0.758 (0.664-0.840)	0.730	0.754	0.700	0.754	0.700
	Cross-validation	0.738 (0.639-0.831)	0.703	0.623	0.800	0.792	0.635
Original texture	Training	0.923(0.868-0.964)	0.847	0.754	0.960	0.958	0.762

model	Cross-validation	0.784 (0.698-0.868)	0.730	0.541	0.960	0.943	0.632
LoG first order model	Training	0.786 (0.698-0.867)	0.748	0.853	0.620	0.732	0.775
	Cross-validation	0.786 (0.698-0.864)	0.730	0.639	0.840	0.830	0.656
LoG texture model	Training	0.733 (0.634-0.828)	0.757	0.574	0.980	0.972	0.653
	Cross-validation	0.817 (0.736-0.891)	0.748	0.623	0.900	0.884	0.662
Wavelet first order model	Training	0.781 (0.692-0.865)	0.730	0.525	0.980	0.970	0.628
	Cross-validation	0.831 (0.747-0.903)	0.793	0.754	0.840	0.852	0.737
Wavelet texture model	Training	0.815 (0.729-0.889)	0.784	0.689	0.90	0.894	0.703
	Cross-validation	0.828 (0.739-0.897)	0.784	0.738	0.840	0.849	0.724
